# Effect analysis of entecavir on serum hyaluronic acid, laminin and IV collagen in the treatment of hepatitis B E-antigen-positive chronic hepatitis B

**DOI:** 10.4314/ahs.v24i4.6

**Published:** 2024-12

**Authors:** Jiancheng Qian, Xiaoyong Sun, Yue Cheng

**Affiliations:** 1 Department of Infectious Disease, Tongde Hospital of Zhejiang Province, Hangzhou, China; 2 Department of Infectious Disease, Xinchang County People's Hospital, Shaoxing, China; 3 Laboratory Medicine, Tongde Hospital of Zhejiang Province, Hangzhou, China

**Keywords:** Entecavir, e antigen-positive chronic hepatitis B, serum hyaluronic acid (HA), laminin (LN), type IV collagen (IVC), clinical outcome analysis

## Abstract

**Background:**

To observe and analyse the clinical effects of entecavir on serum hyaluronic acid (HA), laminin (LN), and type IV collagen (IVC) in patients with hepatitis B e-antigen (HBeAG)-positive chronic hepatitis B during clinical treatment.

**Methodology:**

The patients in the control group received clinical treatment with entecavir monotherapy, while those in the observation group underwent thymalfasin + entecavir combination therapy. The clinical curative effects of immune checkpoint inhibitors at different concentrations on diseases were compared from all aspects.

**Results:**

There were lower levels of total bilirubin (TBIL) and alanine transaminase (ALT) in the observation group, a more satisfactory improvement in immune function-related indicators, and lower levels of HA, LN, and IVC in the observation group, which were statistically different between the two groups (P<0.05). The levels of liver function indicators, immune function-related indicators (CD3+, CD4+, CD8+, CD4+/CD8+, CD4+/CD8+), and HA, LN and IVC were not statistically different between the two groups before treatment.

**Conclusion:**

Entecavir is highly effective in the clinical treatment of HBeAG-positive chronic hepatitis B. However, entecavir + thymalfasin combination therapy can alleviate the clinical symptoms. In this way, liver fibrosis can be prevented in patients with HBeAG-positive chronic hepatitis B, and the clinical curative effect can be enhanced.

## Introduction

The chronic hepatitis B virus (CHB) is the main cause of chronic hepatitis B (CHB)^1^. With no prompt treatment and intervention, CHB may develop into liver cirrhosis and can often lead to a series of such complications as gastrointestinal bleeding and hepatorenal syndrome. In the treatment of cirrhosis and other diseases related to it, thymalfasin is a short titanium therapeutic substance 2. Conversely, entecavir exhibits some antiviral activity, and it is usually prescribed for CHB and other diseases with active pathological changes in clinical liver histology 3. In this context, entecavir was employed in the clinical treatment of hepatitis B e-antigen (HBeAG)-positive CHB in this study so as to observe and analyse the clinical effects of entecavir on serum hyaluronic acid (HA), laminin (LN), and type IV collagen (IVC).

## Patients and methods

### Patients

A total of 236 patients with HBeAG-positive CHB admitted to our hospital from March 2021 to March 2022 were selected using a random number table. They were randomly divided into the control group (n=118) and the observation group (n=118). No statistically significant differences were found in all basic data, including age, gender, and disease duration, between the two groups of patients (P>0.05, Table 1). Hence, these data were worthy of further comparative analysis.

**Table 1:** Basic data [n, (%)] (x̅±s)

Group	Number of subjects	Gender ratio	Age (years)	Mean Age (years)	Duration of disease (years)	Mean duration of disease (years)
Control group	118	64/54	32-61	44.67 ± 2.48	3-11	4.82 ± 1.99
Observation	118	61/57	31-59	44.21 ± 2.66	1-10	4.53 ± 1.85
group						
X^2^	-	0.153	0.067	0.018	0.052	0.116
P	-	0.696	0.795	0.893	0.819	0.734

**Inclusion criteria were as follows:** 1) patients whose disease met the diagnostic criteria for HBeAG-positive CHB specified in the Guidelines of Prevention and Treatment of Chronic Hepatitis B (2019 Version)^4^, that is, the serum HBV-deoxyribonucleic acids (DNAs) > 20,000 IU/mL, and 2 x upper limit of normal (ULN) < alanine transaminase (ALT) < 10 x ULN, 2) those who had positive hepatitis B surface antigen for over half a year, 3) those who were further diagnosed with HBeAG-positive CHB through a series of clinical tests, such as imaging, and 4) those who and whose families had been informed of the relevant contents of this study (therapeutic drugs, treatment steps, etc.), and those who voluntarily participated in this study and signed an agreement with our hospital. This study was carried out upon approval of the Ethics Committee of Tongde Hospital of Zhejiang Province. The article has some limitations, such as the population included in the report is those who are positive for surface antigen B for more than six months, and the treatment effect cannot be evaluated in those who are under six months; this study is a single-center study, and it is not a double-blind study. Therefore, to further validate the results of this study, a multicenter randomized, double-blind study is still needed.

**Exclusion criteria were as follows:** 1) patients with hepatitis D virus (HDV), hepatitis C virus (HCV) or hepatitis A virus (HAV) infections, 2) those complicated with cancerous stem cells, liver cirrhosis or other adverse events, 3) female patients in pregnancy or lactation, 4) those who were indulged in excessive drinking and drug infusion before admission, 5) those complicated with malignant tumors, abnormal liver or kidney function, or other malignant diseases, or 6) those who had allergic reactions or contraindications to the drugs (thymalfasin and entecavir) used in this study.

### Methods

The patients in control group received clinical entecavir (Suzhou Dongrui Pharmaceutical Co., Ltd., Approval No.: NMPA H20100129, Suzhou, China) monotherapy. They orally took entecavir once a day at 0.5 mg per time. Meanwhile, the patients in observation group underwent thymalfasin (SciClone Pharmaceuticals Co., Ltd., Approval No.: NMPA H20120531) + entecavir combination therapy. In the observation group, entecavir was administered in a mode and dosage the same as in the control group. Subcutaneous injections of Thymalfasin were given twice a week at 1.6 mg each time. Both groups of patients underwent two months of clinical treatment.

**Evaluation criteria were as follows:** 1) Evaluation of improvement in liver function-related indexes after treatment: The total bilirubin (TBIL) and ALT in the serum of patients were measured before and after treatment. 2) Evaluation of improvement in immune function-related indexes after treatment: Before and after treatment, peripheral venous blood (5 mL) was sampled from each patient in both groups in the early morning for determination of immune function-related indexes mainly including cluster of differentiation (CD)3+, CD4+, CD8+ and CD4+/CD8+ by flow cytometry. 3) Evaluation of improvement in relevant clinical indexes (HA, LN and IVC) after treatment. Before and after treatment, peripheral venous blood (5 mL) was sampled from each patient in both groups in the early morning and centrifuged for 10 min at 3000 rpm. Later, the serum samples were collected, and the serum levels of HA, LN and IVC were detected via radioimmunoassay.

### Statistical analysis

Statistical Product and Service Solutions (SPSS) 19.0 software (SPSS Inc., Chicago, IL, USA) was used to analyse all data. The measurement data were expressed as (^-^χ±s) and examined by t-test, while the enumeration data were expressed as [n (%)] and analysed by χ^2^ test. P<0.05 represented that the difference was statistically significant.

## Results

### The improvement effect of liver function-related indicators before and after treatment

After treatment, the levels of liver function-related indicators in the control group and the observation group showed a downward trend, and compared with the control group, the levels of TBIL and ALT indicators in the observation group were lower, with statistical differences (P < 0.05), as shown in Table 2.

**Table 2:** Evaluation of the improvement effect of liver function-related indicators before and after treatment (x̅±s)

Group	Number of subjects	Pre-treatment TBIL (µmol/l)	TBIL (µmol/l) after treatment	Pretreatment ALT (U/L)	Post-treatment ALT (U/L)
Control	118	479.64 ± 56.49	97.64 ± 38.16	463.48 ± 173.03	68.64 ± 33.02
group					
Observation	118	485.64 ± 86.25	404.54 ± 98.64	474.96 ± 169.58	114.64 ± 68.26
group					
t	-	0.436	17.896	1.436	2.776
P	-	0.648	<0.001	0.827	<0.001

### The improvement effect of immune function-related indicators before and after treatment

After treatment with different drugs, the levels of CD3 +, CD4 +, and CD4 +/CD8 + indicators in the two groups were increased, while the level of CD8 + hands was decreased. Compared with the control group, the improvement effect of immune function-related indicators in the observation group was more satisfactory after treatment, with statistical significance (P < 0.05), as shown in Table 3.

**Table 3:** Evaluation of the improvement effect of immune function-related indicators before and after treatment (x̅±s)

Metrics	Treatment time	Control (n = 118)	Observation group (n = 118)	t	P
CD3 + (%)	Before treatment	48.52 ± 5.99	49.49 ± 5.26	1.322	0.186
					
	Post Treatment	51.89 ± 5.24	55.29 ± 5.18	5.013	<0.001
CD4 + (%)	Before treatment	27.92 ± 4.49	28.13 ± 4.41	0.363	0.817
	Post Treatment	30.73 ± 4.51	34.31 ± 4.86	5.865	<0.001
CD8 + (%)	Before treatment	25.09 ± 3.36	25.13 ± 2.35	0.106	0.916
	Post Treatment	23.42 ± 2.48	21.11 ± 3.19	6.210	<0.001
CD4 + /CD8 +	Before treatment	1.02 ± 0.36	1.03 ± 0.35	0.216	0.829
					
	Post Treatment	1.43 ± 0.21	1.72 ± 0.33	8.054	<0.001

### The improvement effect of relevant clinical indicators before and after treatment

There was no significant difference in the levels of relevant indicators (HA, LN and IVC) before treatment between the two groups (P > 0.05); after treatment with different drugs, the levels of HA, LN and IVC showed a downward trend, compared with the control group, the levels of the above three indicators in the observation group were lower, with a statistical difference (P < 0.05), as shown in Table 4.

**Table 4:** Assessment of the improvement effect of relevant clinical index levels before and after treatment (x̅±s)

Metrics	Treatment time	Control (n = 118)	Observation group (n = 118)	t	P
HA	Before treatment	258.34 ± 53.31	258.68 ± 53.42	0.049	0.961
	Post Treatment	179.28 ± 42.57	153.14 ± 31.46	5.364	<0.001
LN	Before treatment	173.25 ± 21.27	172.63 ± 21.16	0.225	0.823
	Post Treatment	132.27 ± 22.69	106.29 ± 18.53	9.634	<0.001
IVC	Before treatment	131.25 ± 33.27	130.64 ± 33.16	0.141	0.888
	Post Treatment	119.27 ± 22.69	95.27 ± 20.53	8.520	<0.001

## Discussion

In CHB, the antiviral effect is determined by therapeutic drugs, virus hosts, and viruses, so it may be unsatisfactory for some patients. Clinical data indicate that CHB can progress to liver cirrhosis within five years in over 20% of patients. A relatively large proportion of patients with liver cirrhosis tend to suffer from liver dysfunction or hepatocellular carcinoma^4^,^5^. In CHB patients, clinical scientific and reasonable antitoxic and antivirus treatments are effective in alleviating the damage to the liver tissues, which reduces the risk of hepatitis progressing to serious diseases like liver cirrhosis and cancer^6^. It has been clinically shown that the continuous development of CHB will lead to the diffusive growth and development of fiber structure in liver tissues, damaging liver tissue structure and affecting blood supply and circulation^7^.

Entecavir, a drug used in the treatment of CHB, can not only evidently inhibit the polymerase of HBV but also effectively control the production of reverse transcription negative strands of pre-genome messenger RNAs, thus further improving the levels of indexes related to human liver histology^8^. According to the research and exploration of Wu IC, Liu WC et al.^9^, the discontinuation of treatment of this therapeutic drug increases the recurrence rate of the disease, showing a bad prognosis. Therefore, entecavir + thymalfasin combination therapy was employed, which achieved significant clinical effects. It is because thymalfasin, which is separated from thymosin, can facilitate the growth and development of lymphocytes, increase the production and expression of many cytokines, such as interleukins, and increase the body's immunity to viruses, thus improving immunity and alleviating clinical symptoms^9^-^11^.

The results of this study revealed that after treatment, the observation group had a higher overall response rate than the control group. In addition, the improvement in immune function-related indexes was more apparent, and the levels of HA, LN, and IVC were lower in the observation group than in the control group. It can be concluded that entecavir + thymalfasin combination therapy can notably alleviate the clinical symptoms of CHB patients and increase their immunity and resistance. Moreover, liver fibrosis is a pathological development stage of multiple chronic liver diseases treated clinically. The relevant index, serum HA, is produced by interstitial cells, which can clearly present liver fibrosis's development and pathological changes. IVC, a fibrous glycoprotein, will proliferate in the case of human liver fibrosis, thereby seriously damaging the liver. The LN level exceeding the average value indicates the occurrence of liver fibrosis, which further results in diffusive liver tissue injuries. Entecavir + thymalfasin combination therapy effectively controls the expression levels of serum HA, IVC, and LV and can repair the damaged liver cells, thus achieving anti-fibrosis in the clinic. This way, the clinical curative effect can be enhanced in patients with HBeAG-positive CHB. It was found in this study that the expression levels of serum HA, IVC and LV remarkably declined after entecavir + thymalfasin combination therapy, suggesting that the combination therapy can effectively control the development of liver fibrosis by lowering the expression levels of serum HA, IVC, and LV. In treating CHB, entecavir can effectively manage the production of DNA polymerases induced by high concentrations of phosphorylated components in cells. Using lower-concentration entecavir can not only limit HBV replication, but also reduce cytotoxicity, which is very crucial for controlling the speed of HBV and DNA replication. Furthermore, entecavir can effectively reduce the damage of viruses in serum tissues and liver tissues to the body, protect newly generated liver cells and nearby liver cells against virus infection to the greatest extent, and reduce the inflammation and necrosis of liver tissues, which confirms the practicability of the drug in the treatment of CHB.

The article has some limitations, such as the population included in the report is those who are positive for surface antigen B for more than six months, and the treatment effect cannot be evaluated in those who are under six months; this study is a single-center study, and it is not a double-blind study. Therefore, to further validate the results of this study, a multicenter randomized, double-blind study is still needed

## Conclusion

In conclusion, entecavir's clinical efficacy in treating chronic hepatitis B disease is remarkable. Still, when discontinued, it may lead to an increase in relapse rates and a less favourable prognosis. Using entecavir in combination with other drugs can improve the patient's clinical symptoms and significantly reduce serum HA, IVC, and LV levels, thereby inhibiting the progression of liver fibrosis.

## Data Availability

The datasets used and analysed during the current study are available from the corresponding author on reasonable request.
